# Assessing the Justification, Funding, Success, and Survival Outcomes of Randomized Noninferiority Trials of Cancer Drugs: A Systematic Review and Pooled Analysis

**DOI:** 10.1001/jamanetworkopen.2019.9570

**Published:** 2019-08-30

**Authors:** Bishal Gyawali, Frazer A. Tessema, Emily H. Jung, Aaron S. Kesselheim

**Affiliations:** 1 Division of Cancer Care and Epidemiology, Departments of Medical Oncology and Public Health Sciences, Queen’s University, Kingston, Ontario, Canada; 2 Program on Regulation, Therapeutics, and Law (PORTAL), Division of Pharmacoepidemiology and Pharmacoeconomics, Brigham and Women’s Hospital, Harvard Medical School, Boston, Massachusetts

## Abstract

**Importance:**

Noninferiority trials test whether a new intervention is not worse than the comparator by a given margin.

**Objectives:**

To study the characteristics of published randomized noninferiority trials in oncology with overall survival as an end point, to assess the association of justification and success in achieving noninferiority with the funding of these trials, and to evaluate the association of such trials with patient survival.

**Data Sources:**

A systematic search of PubMed and Google Scholar databases was conducted in March 2018, with no date restrictions.

**Study Selection:**

Randomized noninferiority trials of cancer drug therapies with overall survival as an end point were included. Trials of decision support, supportive care, and nondrug treatment in both arms were excluded.

**Data Extraction and Synthesis:**

This study followed the Preferred Reporting Items for Systematic Reviews and Meta-analyses (PRISMA) guidelines for meta-epidemiological studies. Studies were screened for eligibility criteria, and data on criteria for noninferiority, funding, success (achieving noninferiority), and hazard ratios with confidence intervals for overall survival were extracted. Hazard ratios for overall survival were pooled across trials using a random-effects model.

**Main Outcomes and Measures:**

Associations of the justification for using a noninferiority design and success in achieving noninferiority with the source of funding were assessed. Overall pooled hazard ratios and confidence intervals for overall survival were calculated.

**Results:**

Among 74 noninferiority trials of cancer drug therapies, 23 (31%; enrolling 21 437 patients) used overall survival as the primary end point. The noninferiority margins for the hazard ratio of overall survival ranged from 1.08 to 1.33. Noninferiority design was justified in 14 trials (61%) but not in 9 (39%). Overall, 18 trials (78%) concluded with a finding of noninferiority. Industry funding was associated with lack of justification for noninferiority design (*P* = .02, assessed using the Fisher exact test) but not with success in proving noninferiority (*P* = .80, assessed using the Fisher exact test). When the hazard ratios across the trials were pooled, there was no beneficial or detrimental association with overall survival, with a pooled hazard ratio of 0.97 (95% CI, 0.92-1.02).

**Conclusions and Relevance:**

The findings suggest that a substantial fraction of noninferiority trials in oncology, most of which are industry funded, lack justification for such a design. Greater attention to the use of noninferiority designs in randomized clinical trials of cancer drugs from local and national regulators is warranted.

## Key Points

**Question:**

Is the source of funding associated with the justification for using noninferiority design and success in claiming noninferiority in cancer drug trials, and are these trials associated with changes in patient survival?

**Findings:**

In this systematic review and pooled analysis of 23 randomized noninferiority trials of cancer drugs, which used overall survival as the end point and enrolled 21 437 patients, industry funding was associated with lack of justification but not with success in achieving noninferiority. No association of noninferiority trials with patient survival was found.

**Meaning:**

Greater regulatory attention should be paid to randomized noninferiority trials of cancer drugs, especially regarding the justification for using such a design.

## Teaser

This systematic review and pooled analysis of randomized noninferiority trials of cancer drugs assesses the association of justification for using noninferiority design and success in achieving noninferiority with the funding of these trials and the association of these trials with patient survival.

## Introduction

Randomized clinical trials designed with a noninferiority hypothesis test whether the experimental treatment is worse than the standard of care by a given margin called the *noninferiority margin*.^1^ Noninferiority trials are useful to test interventions that aim at providing compensatory benefits without necessarily compromising efficacy. If the new drug or strategy reduces cost or adverse effects or improves the ease of administration, patients may reasonably accept the possibility of slight reductions in efficacy.

However, there are numerous concerns associated with defining, designing, conducting, reporting, and interpreting noninferiority trials,^1,2^ including in oncology.^3^ For example, how much of a possible reduction in efficacy is reasonable? How noninferiority limits are defined may raise ethical issues related to the sufficiency of patient informed consent.^4^ In some cases, noninferiority designs have been useful. For example, the standard of care for the use of zoledronic acid for preventing skeletal-related events in patients with cancer and bone metastases used to be an injection every month. Noninferiority trials have now established that the frequency could safely be reduced to once every 3 months without compromising efficacy outcomes.^5,6^

Noninferiority trials have been applied sparingly to oncology drugs because patients with cancer are unlikely to accept even the possibility of reduced efficacy of their oncology drugs. However, in a 2018 case, a noninferiority trial served as the pivotal trial for US Food and Drug Administration approval of a new cancer drug, even though the new drug did not necessarily provide any benefits in terms of cost, ease of administration, or toxic effects.^7^ Previous studies have shown that industry-funded trials were more likely to use a noninferiority design than nonindustry-sponsored trials.^8^

In cancer treatment, noninferiority trials with overall survival (OS) outcomes are most critical from a clinical and regulatory point of view because it would be unwise to use a new therapy based on its noninferiority regarding response rates or progression-free survival without knowing the effects on OS, as the drug might very well have more substantially inferior outcomes on survival. Similarly, patients are most concerned with compromise in survival when the results from noninferiority trials are translated in clinics.

To understand the characteristics of noninferiority trials used in evaluating cancer drugs, we sought to systematically review the use of the noninferiority hypothesis in randomized clinical trials in the field of oncology. In this systematic review and pooled analysis, we investigated the basis or reasoning for using noninferiority designs, the funding of these trials, and efficacy outcomes.

## Methods

The objectives of this study were as follows: (1) to study the characteristics of randomized cancer drug trials that use noninferiority design, including the success rate and justification for using noninferiority designs, (2) to study whether the success in claiming noninferiority or the lack of justification for using noninferiority design was associated with the source of funding, and (3) to study the overall association of the drug being tested with patients’ survival. This study was conducted in accordance with the modification of Preferred Reporting Items for Systematic Reviews and Meta-analyses (PRISMA) reporting guideline for meta-epidemiological studies.^9^

### Study Identification

We conducted a systematic search of the PubMed database in March 2018 without date restrictions, supplemented with a search of the Google Scholar database. To identify published trials in cancer that used a noninferiority design, we used the search terms *neoplasms* or *cancer* or tumo* or malignan* or *oncology* AND non inferior* or non-inferior* or noninferior* and limited our search to findings published in English. After title and abstract screening by 2 of us (B.G. and F.A.T.) acting independently, the full texts of potentially relevant studies were downloaded and reviewed for the following exclusion criteria: (1) not a randomized design; (2) trials in pediatric populations; (3) trials of surgery or radiotherapy only in all treatment arms (trials comparing a drug in 1 arm and surgery or radiotherapy in other arms were eligible); (4) trials of decision support, diagnostic modalities, or supportive care; (5) trials assessing behavioral interventions, genetic counseling, screening, or diagnostic modalities; (6) trials assessing only pharmacokinetics; and (7) post hoc analyses. For this study, we limited our analysis to trials with a primary or a coprimary end point of OS for 2 reasons. First, the most important concern for patients making treatment decisions based on noninferiority trials is the potential for compromise in survival, rather than compromise in surrogate measures. Second, we planned to conduct a pooled analysis, and it would not be possible to pool survival with other surrogate measures across the trials.

### Data Extraction

Data were independently extracted from published reports by 2 of us (F.A.T. and E.H.J) and verified by a third author (B.G.), with discrepancies resolved through consensus of all authors. We collected year of publication, treatment setting, primary end point, sample size, blinding (ie, double blind or open label), funding (ie, public, industry, or mixed public and industry), and the authors’ listed criteria for using a noninferiority design.

We judged whether the noninferiority design was justified for each trial. Noninferiority was considered justified if the intervention being tested provided at least 1 of the following benefits to patients: (1) less cost; (2) decreased frequency of administration; (3) increased ease of administration, such as noninjectable (eg, oral) vs injectable formulation; or (4) improved quality of life.

We also extracted information on the outcome of the trial in terms of whether noninferiority was achieved. A trial was considered successful if it achieved noninferiority based on its own criteria. For trials that achieved noninferiority, we checked if the intervention also proved superiority based on 95% CIs and whether the publication concluded superiority. For quality-of-life outcomes, we considered quality of life to be improved if the summary was statistically better; we did not examine each domain of the assessment tool separately. Finally, information on the hazard ratio (HR) and 95% CI for OS were extracted from the published reports for pooled analysis. If a CI different from a 95% CI was reported, we recalculated the CI to a 95% CI.

### Statistical Analysis

The associations of the justification for using the noninferiority design and success in achieving noninferiority with the funding source were assessed using Fisher exact tests. The overall association of the trial drugs on OS was assessed by pooling the HRs across the trials using a random-effects meta-analysis to account for heterogeneity. Heterogeneity among studies was assessed using the Cochrane *Q* statistic (assumption of homogeneity was considered invalid for values of *P* < .10) and quantified using an *I*^2^ test. Subgroup analyses were prespecified and included funding, blinding, and success. All statistical analyses were conducted using Stata version 15 (StataCorp), and a 2-sided *P* < .05 was considered statistically significant.

## Results

**Figure 1.**
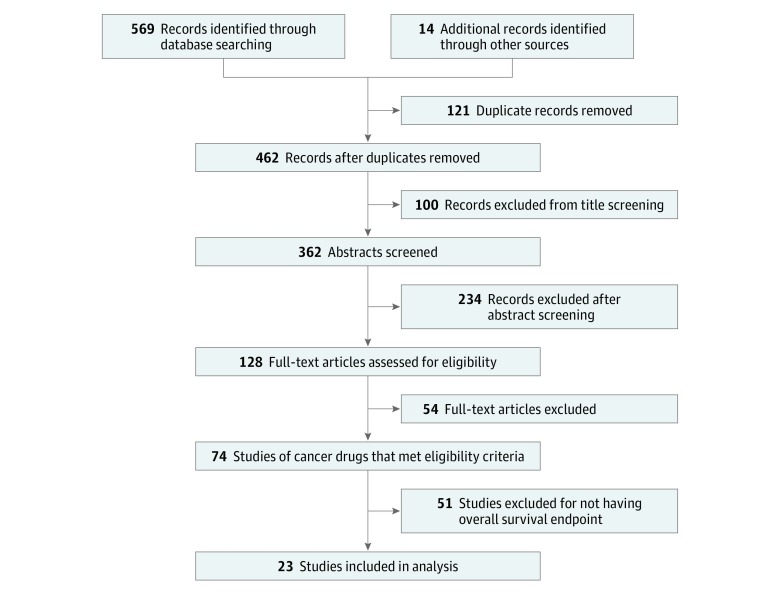
Flow Diagram for the Selection of Studies

Among 128 randomized noninferiority trials in oncology in adults identified through our search, 74 (58%) were drug trials. Of those, 23 (31%) enrolled 21 437 patients, used OS as the primary or coprimary end point, and were therefore included in our analysis (Figure 1 and Table 1).^10,11,12,13,14,15,16,17,18,19,20,21,22,23,24,25,26,27,28,29,30,31,32^ None of these trials were blinded. Approximately half (12 [52%]) had industry funding, 5 (22%) were publicly funded, and 6 (26%) had mixed funding.

**Table 1.** Justifications for Noninferiority Trials of Cancer Drugs in Oncology With Overall Survival Endpoint

Source	Total Participants, No.	Cancer Type	Patients, No.		QoL Results	Justification of Noninferiority Design
			Experimental Arm	Control Arm		
Adams et al,^10^ 2011	2445	Colorectal	815	815	Better	Justified: intermittent vs continuous oxaliplatin
Boku et al,^11^ 2009	468	Gastric	234	234	Not assessed	Justified: oral S-1 vs IV 5-FU; S-1 less expensive
Crook et al,^12^ 2012	1386	Prostate	690	696	Better	Justified: intermittent vs continuous androgen deprivation therapy
daSilva et al,^13^ 2014	918	Prostate	462	456	Not statistically different	Justified: intermittent vs continuous androgen deprivation therapy
Ferry et al,^14^ 2017	909	NSCLC	453	456	Not different; statistical comparison not provided	Justified: carboplatin vs cisplatin
Fink et al,^15^ 2012	795	SCLC	346	334	Not assessed	Not justified: topotecan-cisplatin vs cisplatin-etoposide
Hofheinz et al,^16^ 2012	392	Colorectal	195	197	Not assessed	Justified: oral capecitabine vs IV 5-FU
Hussain et al,^17^ 2013	1535	Prostate	770	765	Slightly better but summary statistic not available	Justified: intermittent vs continuous hormone therapy
Kehoe et al,^18^ 2015	500	Ovarian	274	276	Not statistically different	Justified: avoiding surgery
Kim et al,^19^ 2008	1433	NSCLC	723	710	Better	Justified: oral gefitinib vs IV docetaxel
Kim et al,^20^ 2009	491	Colorectal	246	245	Not reported	Not justified: FOLFOX vs irinotecan
Kitagawa et al,^21^ 2015	253	Cervical	126	127	Better	Justified: carboplatin vs cisplatin; less hospitalization time
Kubota et al,^22^ 2015	608	NSCLC	301	295	Better	Justified: oral S-1 vs IV docetaxel in combination with platinum
Kudo et al,^23^ 2018	1492	Liver	476	475	Not statistically different	Not justified: lenvatinib vs sorafenib; lenvatinib costs more than sorafenib and offers no other benefits
Lang et al,^24^ 2013	564	Breast	279	285	Not statistically different	Not justified: bevacizumab plus capecitabine vs bevacizumab plus paclitaxel; capecitabine and paclitaxel are both established as first-line agents; the control arm of bevacizumab plus paclitaxel is now withdrawn by the FDA
Okamoto et al,^25^ 2010	564	NSCLC	282	281	Not statistically different	Justified: oral S-1 vs IV paclitaxel in combination with carboplatin
Popov et al,^26^ 2008	1921	Colorectal	952	969	Not assessed	Not justified: IV ralitrexed vs IV 5-FU
Price et al,^27^ 2016	1010	Colorectal	596	594	Not assessed	Not justified: panitumumab vs cetuximab
Satouchi et al,^28^ 2014	284	SCLC	142	142	Not statistically different	Not justified: amrubicin-platinum vs irinotecan-platinum
Scagliotti et al,^29^ 2008	1725	NSCLC	862	863	Not assessed	Not justified: cisplatin-pemetrexed vs cisplatin-gemcitabine; pemetrexed offered a better profile for some adverse effects but was much more expensive than gemcitabine
Shitara et al,^30^ 2017^a^	741	Gastric	246 and 247	248	Statistical comparison not provided	Not justified: abraxane 3 times per week vs abraxane weekly vs paclitaxel weekly; abraxane costs more than 50-fold what paclitaxel costs; premedication not necessary with abraxane
Takashima et al,^31^ 2016	618	Breast	309	309	Better	Justified: oral S-1 vs IV taxane
Uesaka et al,^32^ 2016	385	Pancreas	192	193	Better	Justified: oral S-1 vs IV gemcitabine

Abbreviations: FDA, US Food and Drug Administration; FOLFOX, 5-fluorouracil plus leucovorin plus oxaliplatin; 5-FU, 5-fluorouracil; IV, intravenous; NSCLC, non–small cell lung cancer; QoL, quality of life; SCLC, small cell lung cancer.

^a^ The trial by Shitara et al^30^ had 3 arms.

Noninferiority was defined in terms of the upper limit of the CI of the HR for OS in 20 (87%) trials. Most trials used 95% CIs,^11,12,13,14,20,22,23,24,25,26,28,29,30,31,32^ but some used 90% CIs,^17,18,21^ 96% CIs,^19^ and 80% CIs^10^ (Table 2). A 2012 trial^16^ defined noninferiority in terms of difference in 5-year OS rates, a 2016 trial as retaining at least 50% of the OS effects of the control arm,^27^ and a 2012 trial in terms of the lower limit of the 95% CI for median OS greater than median minus 10% of the overall survival of the control arm^15^ (Table 2).

**Table 2.** Outcomes of Noninferiority Trials of Cancer Drugs in Oncology With OS End Point

Source	Cancer Type	Funding	Noninferiority Criteria for Hazard Ratio	Success	OS, HR (95% CI)
Adams et al,^10^ 2011^a^	Colorectal	Mixed	1.162^b^	No	1.08 (1.01-1.17)
Boku et al,^11^ 2009	Gastric	Mixed	1.16^c^	Yes	0.83 (0.68-1.01)
Crook et al,^12^ 2012	Prostate	Public	1.25^c^	Yes	1.02 (0.86-1.21)
daSilva et al,^13^ 2014	Prostate	Public	1.21^c^	Yes	0.90 (0.76-1.07)
Ferry et al,^14^ 2017	NSCLC	Mixed	1.2^c^	Yes	0.93 (0.83-1.04)
Fink et al,^15^ 2012	SCLC	Industry	Lower limit of 95% CI of median OS should be >10% less than the median OS of control arm	Yes	0.93 (0.79-1.10)
Hofheinz et al,^16^ 2012	Colorectal	Industry	5-y OS rate difference 12.5%	Yes	0.67 (0.44-1.00)
Hussain et al,^17^ 2013^a^	Prostate	Public	1.2^d^	No	1.10 (0.99-1.23)
Kehoe et al,^18^ 2015^a^	Ovarian	Public	1.18^d^	Yes	0.87 (0.72-1.05)
Kim et al,^19^ 2008^a^	NSCLC	Industry	1.154^e^	Yes	1.02 (0.91-1.15)
Kim et al,^20^ 2009	Colorectal	Industry	1.33^c^	Yes	0.92 (0.80-1.10)
Kitagawa et al,^21^ 2015^a^	Cervical	Public	1.29^d^	Yes	0.99 (0.79-1.25)
Kubota et al,^22^ 2015	NSCLC	Industry	1.322^c^	Yes	1.01 (0.84-1.23)
Kudo et al,^23^ 2018	Liver	Industry	1.08^c^	Yes	0.92 (0.79-1.06)
Lang et al,^24^ 2013	Breast	Mixed	1.33^c^	No	1.06 (0.80-1.40)
Okamoto et al,^25^ 2010^a^	NSCLC	Industry	1.33^c^	Yes	0.93 (0.69-1.17)
Popov et al,^26^ 2008	Colorectal	Industry	1.25^c^	No	1.01 (0.84-1.23)
Price et al,^27^ 2016	Colorectal	Industry	≥50% Retention of OS effects of control	Yes	0.94 (0.82-1.07)
Satouchi et al,^28^ 2014	SCLC	Industry	1.31^c^	No	1.43 (1.10-1.85)
Scagliotti et al,^29^ 2008	NSCLC	Industry	1.176^c^	Yes	0.94 (0.84-1.05)
Shitara et al,^30^ 2017^f^	Gastric	Industry	1.25^c^	Yes for arm A; no for arm B	Arm A, 0.97 (0.76-1.23); arm B, 1.06 (0.87-1.31)
Takashima et al,^31^ 2016	Breast	Mixed	1.333^c^	Yes	1.05 (0.86-1.27)
Uesaka et al,^32^ 2016	Pancreas	Mixed	1.25^c^	Yes	0.57 (0.44-0.72)

Abbreviations: HR, hazard ratio; NSCLC, non–small cell lung cancer; OS, overall survival; SCLC, small cell lung cancer.

^a^ Reported a CI different from a 95% CI, which was recalculated in the Table to 95% CIs.

^b^ Noninferiority criteria defined by upper limit of 80% CI for HR.

^c^ Noninferiority criteria defined by upper limit of 95% CI for HR.

^d^ Noninferiority criteria defined by upper limit of 90% CI for HR.

^e^ Noninferiority criteria defined by upper limit of 96% CI for HR.

^f^ The trial by Shitara et al^30^ had 3 arms.

The upper limit of the CI for the HR for OS in the 20 trials that used that measure as their criteria for noninferiority ranged from 1.08 to 1.33. Of these, 14 trials (70%)^12,13,14,17,20,21,22,24,25,28,30,31,32^ had a limit of 1.2 or more, meaning that up to a 20% increase in mortality risk was considered noninferior (ie, acceptable). A 2017 trial^30^ had 3 arms comparing 2 different regimens in noninferiority design against the control.^30^ Quality of life was improved in 8 trials (35%),^10,12,17,19,21,22,31,32^ not improved in another 8 (35%),^13,14,18,23,24,25,28,30^ and not assessed or assessed but not reported in the remaining 7 (30%) (Table 1).^11,15,16,20,26,27,29^

### Justification for Noninferiority

Overall, 14 trials (61%) met our criteria for justifying the noninferiority design (Table 1).^10,11,12,13,14,16,17,18,19,21,22,25,31,32^ A new oral drug vs injectable standard was the most common justification (7 trials)^11,16,19,22,25,31,32^ for using the noninferiority design, followed by intermittent vs continuous therapies (4 trials).^10,12,13,17^ Other justifications included avoiding surgery with chemotherapy^18^ or using carboplatin instead of cisplatin.^14,21^

Among the 9 noninferiority trials (39%) that did not meet our justification criteria, comparisons were made between oral-oral or intravenous-intravenous drugs, usually of the same class. Most trials^15,20,23,26,27,28,29,30^ (8 [89%]) that did not meet any justification for noninferiority were industry funded, while 1 trial^24^ had mixed funding. All publicly funded noninferiority trials met the criteria for justification. Lack of justification was associated with funding source (*P* = .02, assessed using the Fisher exact test).

### Success in Establishing Noninferiority

A total of 18 trials (78%) established noninferiority for the intervention drug (Table 2).^11,12,13,14,15,16,18,19,20,21,22,23,25,27,29,30,31,32^ One trial^32^ proved superiority. Of the 12 industry-funded trials, 10 established noninferiority for the drug being tested.^15,16,19,20,22,23,25,27,29,30^ Success in achieving noninferiority was not associated with funding (*P* = .80, assessed using the Fisher exact test).

### Association With OS

**Figure 2.**
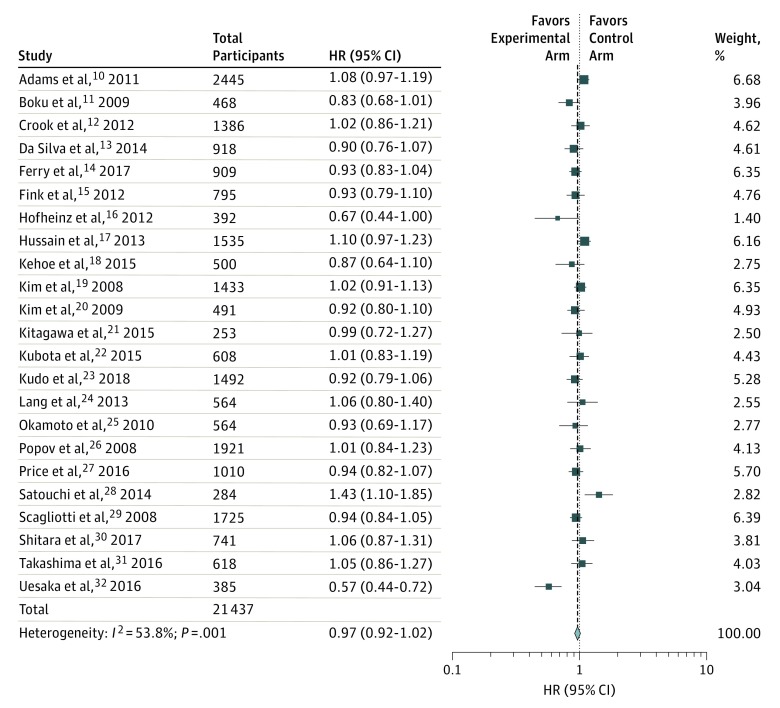
Pooled Analysis of Hazard Ratios (HRs) of Overall Survival Weights are determined from random-effects analysis. The size of each box represents the weight by random-effects method of the contribution of each study to the weight of the sample in meta-analysis. The vertical dashed line indicates the point of summary HR, and the diamond indicates the 95% CI for the summary HR. Hazard ratio values less than 1 reflect protective effects of treatment, and HR values greater than 1 reflect detrimental effects of treatment on survival.

The HR was more than 1 in 10 trials (41%),^10,12,17,19,22,24,26,28,30,31^ but was significant in only 1 trial^28^ (Table 2). When the HRs across trials were pooled using random-effects meta-analysis, there was no beneficial or detrimental association with patient survival (pooled HR, 0.97; 95% CI, 0.92-1.02); the heterogeneity among the trials was substantial as trials across different tumor types were pooled (*I*^2^ = 53.8%, *P* = .001) (Figure 2). This analysis was repeated using comparisons for each cohort of the trial by Shitara et al^30^ (the trial with 3 arms) independently and results did not change. On subgroup analysis, there was no difference between industry-funded trials vs mixed or publicly funded trials as well as no difference between trials in which noninferiority design was justified vs not justified.

## Discussion

In systematically searching for noninferiority trials of cancer drugs, we found that only 31% used OS as the primary end point. Among these trials, the criteria to define noninferiority varied from 1.08 to 1.33 for the upper limit of the CI of the HR for death. Ease of administration (oral formulation vs injectable control drugs) was the most common justification for the noninferiority design, while we found insufficient justification for 40% of such trials. Most trials were successful in proving noninferiority, and when the hazard ratio was pooled across these trials, there was no detrimental effect on OS.

Noninferiority trials in oncology have previously been criticized for “critical deficiencies in design and reporting.”^33^ In this cohort, OS was the primary end point in fewer than one-third of noninferiority trials. However, using a noninferiority hypothesis implies a willingness to compromise efficacy outcomes to achieve benefits in other areas. Surrogate measures, such as progression-free survival, have been shown to overestimate (or, in the case of immunotherapy drugs, underestimate^34^) benefit and translate to smaller than expected gains in OS. In most cases these surrogate measures have been shown to lack correlation with OS.^35^ Thus, a trial testing noninferiority in a surrogate measure is ethically challenging if it is impossible to estimate the magnitude of potential compromise in efficacy and communicate that clearly to patients participating in such trials as part of the informed consent process. Investigators should therefore be wary of conducting trials assessing noninferiority on surrogate measures.

The criteria to define noninferiority among the trials in the cohort varied from 1.08 to 1.33 for the upper bound of the CI of the HR for OS, which means from an 8% to a 33% increase in the hazard of death was considered acceptable (noninferior) in these trials. Furthermore, in multiple cases, this upper limit was defined not for a 95% CI but for a 90% or even an 80% CI. As previous studies have shown, there are no set methods for determining limits defining noninferiority.^3,33^ We also found none of the 4 key justifications—lower toxic effects, lower cost, ease of administration, or better quality of life—for using noninferiority designs in approximately 40% of the cohort. Such benefits should be the primary rationale for a patient consenting to participate in a trial testing if a new treatment is not worse than the standard treatment by a prespecified margin.

Our final objective was to assess if patients enrolled in the experimental arm of noninferiority trials experienced reduced OS compared with those in the control arms. Reassuringly, we found no such association. However, this pooled result should be interpreted with caution because, when examining individual trials, we found 10 trials (43%) in which the HR was more than 1, including 1 in which the HR was significantly more than 1. Similarly, 1 noninferiority trial actually proved superiority for the experimental arm. Subgroup analyses revealed no differences in OS based on funding or reporting 1 of the 4 key justifications for noninferiority.

Half of the trials in our study had industry funding, and having industry funding was significantly associated with missing the justifications we identified for the noninferiority design. Industry-funded noninferiority trials also were successful in proving noninferiority in 83% of cases, although the association of success with funding was not significant. Our results are similar to those of a previous analysis^4^ showing that 43% of noninferiority trials in oncology were industry funded, 73% reported positive results, and OS was the primary end point in 25%. Another study^36^ that included all noninferiority trials across disciplines showed that 83% produced favorable results irrespective of funding source, similar to our findings. These results should be interpreted in light of the fact that success in achieving noninferiority depends not only on the intervention but also on the criteria used to define noninferiority, which is often arbitrary. Furthermore, we cannot rule out publication bias against noninferiority trials that failed to achieve noninferiority.

These findings are important for those helping design and oversee the conduct of noninferiority trials in oncology. Noninferiority trials may be attractive because of the high probability of success.^8^ Indeed, the noninferiority design has been described as having a low risk of failure.^36^ However, our data show that institutional review boards and drug regulators should take an active role in adjudicating whether the noninferiority design is acceptable for the given question. When noninferiority design trials are considered important, the criteria to define noninferiority should be clearly defined based on a widely accepted rationale and should incorporate patient input.

The Consolidated Standards of Reporting Trials (CONSORT) statement on reporting noninferiority trials recommends providing a rationale for the noninferiority design and the criteria for defining noninferiority^37^; however, further research is needed to assess if this recommendation has improved reporting practices in oncology. Similarly, the US Food and Drug Administration has issued a guidance for industry on noninferiority trials. However, the US Food and Drug Administration recommends a noninferiority design be chosen “when it would not be ethical to use a placebo.”^38^ While that is necessary, it is not sufficient, and superiority design trials against an active comparator should be encouraged unless the noninferiority design is justified for other compelling reasons, such as the ones we have mentioned. The guidance could also be improved by highlighting the need to incorporate patient input on the acceptable margin for defining noninferiority among various tumor types.

### Limitations

This study has limitations. Although our analysis included trials involving more than 21 000 participants, for the analysis of the association of different parameters with funding, this study was limited by the relatively small total number of trials (23). Another important limitation is our consensus-based definition for adjudicating whether the noninferiority design was justified. However, other authors have also concluded that “any new treatment, even if noninferior to standard treatment, should have some benefits, such as for quality of life, cost, or safety.”^11^ We focused on noninferiority trials testing OS end points; however, many trials also test noninferiority in surrogate measures, such as response rates. The association of such trials with patient treatment outcomes is a topic for future research.

## Conclusions

Noninferiority randomized trials in oncology should be used only when there are important potential benefits that the experimental drug can offer patients. However, among 23 such trials testing OS, we found that a substantial fraction did not offer any of the 4 key criteria for justification. Greater attention to the use of noninferiority designs in cancer drug clinical trials from local and national regulators is warranted.
